# Hydroleine as a Tissue-Builder

**Published:** 1905-03

**Authors:** 


### HYDROLEINE AS A TISSUE BUILDER.

Every physician has at sometime in his professional career, become discouraged in his ability to treat successfully cases of malnutrition, wasting diseases, and kindred ailments that failed to respond to the ordinary tonic treatment so much in vogue among us.

I would invariably place my patients suffering from debility, bronchitis, and all diseases of a wasting nature, on a system of tonic treatment, which I found gave me uncertain results, and in looking about for an improvement on the old method of temporary stimulation, I was led to give Hydroleine a fair and impartial trial. The results obtained were so encouraging that I have learned to trust it in all cases where I desire a permanent tissue builder and reconstructive. I find that it is agreeable to the patient, well borne by the stomach, and the only combination containing cod-liver oil that does not produce unpleasant eructations, so objectionable in most cod-liver oil preparations. It is a pancreatized, predigested oil, giving no extra work to the digestive apparatus, being ready for alimentation as soon as ingested.—*W. Harpur Sloan, M.D., Chief of Ear Department, Medico-Chirurgical Hospital, Philadelphia, Pa.*

